# High spatio-temporal variability in Acroporidae settlement to inshore reefs of the Great Barrier Reef

**DOI:** 10.1371/journal.pone.0209771

**Published:** 2019-01-30

**Authors:** Johnston Davidson, Angus Thompson, Murray Logan, Britta Schaffelke

**Affiliations:** Australian Institute of Marine Science, Townsville, Queensland, Australia; University of Barcelona, SPAIN

## Abstract

Recovery of coral reefs after disturbance relies heavily on replenishment through successful larval settlement and their subsequent survival. As part of an integrated study to determine the potential effects of water quality changes on the resilience of inshore coral communities, scleractinian coral settlement was monitored between 2006 and 2012 at 12 reefs within the inshore Great Barrier Reef. Settlement patterns were only analysed for the family Acroporidae, which represented the majority (84%) of settled larvae. Settlement of Acroporidae to terracotta tiles averaged 0.11 cm^**-2**^, representing 34 ± 31.01 (mean ± SD) spat per tile, indicating an abundant supply of competent larvae to the study reefs. Settlement was highly variable among reefs and between years. Differences in settlement among locations partly corresponded to the local cover of adult Acroporidae, while substantial reductions in Acroporidae cover caused by tropical cyclones and floods resulted in a clear reduction in settlement. Much of the observed variability remained unexplained, although likely included variability in both connectivity to, and the fecundity of, adult Acroporidae. The responsiveness of settlement patterns to the decline in Acroporidae cover across all four regions indicates the importance of supply and connectivity, and the vulnerability towards region-wide disturbance. High spatial and temporal variability, in addition to the resource-intensive nature of sampling with settlement tiles, highlights the logistical difficulty of determining coral settlement over large spatial and temporal scales.

## Introduction

Replenishment of corals through recruitment (here defined as successful settlement and metamorphosis of newly settled corals, hereafter “spat”, followed by post-settlement survival and growth) is critical for the long-term resilience of reef communities facing exposure to pressures such as thermal bleaching, extreme weather events, outbreaks of coral predators such as the crown-of-thorns starfish (currently referred to as *Acanthaster* cf *solaris*), and disease [1, 2]. Reefs close to the coast are subject to discharge from river systems, exposing coral communities to additional pressures such as increased turbidity, sedimentation, nutrient enrichment, pollutants and hyposalinity [3–6]. Examples world-wide show these pressures are exacerbated by coastal and catchment development [7, 8]. However, while pressures associated with land runoff may affect all stages of the coral life-cycle, the early phases culminating in recruitment of juveniles have been identified as particularly vulnerable [5, 9–11].

The terms settlement and recruitment are sometimes used interchangeably, but in this study, as others ([12–14]), we distinguish between these two life-cycle stages. Successful settlement of coral requires a survivable pathway between viable brood-stock and a substrate onto which coral larvae can metamorphose and develop. Successful recruitment of coral requires the subsequent development of the settled coral spat through juvenile stages towards adulthood. This study focused on the abundance and patterns of coral spat settlement at inshore coral reefs over several years, and environmental factors that may have influenced the variability observed.

Laboratory and field studies show that the elevated concentrations of nutrients, agrochemicals, and turbidity typical of inshore environments can directly affect one or more early stages of development including: gametogenesis, egg size, fertilisation, planulation, and embryo development in corals [5, 15–19]. Higher nutrient availability can also increase the abundance of macroalgae [4, 20] which, through allelochemical and mechanical interactions, can suppress gamete development [21], and reduce settlement [22–24]. High levels of sedimentation, considered both as rate of deposition and the level of accumulation on a surface, can deter coral planulae from settling [25–28], disrupt attachment and metamorphosis processes [29], and smother newly settled corals [3, 13, 30, 31]. Any of these water quality-related pressures on the early life stages of corals have the potential to suppress recovery of coral communities from disturbance events, and increase the likelihood of long-term degradation.

The Reef Plan Marine Monitoring Program (MMP) was initiated in 2005 to monitor the effectiveness of the Reef Water Quality Protection Plan, an initiative of the Australian and Queensland State governments to mitigate detrimental impacts of land-based runoff on the health and resilience of the Great Barrier Reef (GBR) [32]. Given the importance of the recruitment process to the resilience of coral communities, settlement of coral spat was included in the MMP as an indicator of the cumulative success of the processes of gametogenesis, fertilisation, larval survival, settlement, metamorphosis, and early post-settlement survival. The purpose of the analyses presented here was to:

1. Explore the relationship between environmental conditions and coral settlement at 12 nearshore reefs spanning 6.5 degrees of latitude,
2. Contribute new baseline data to the currently spatially and temporally constrained information on coral settlement to inshore habitats of the GBR [33–39].

## Materials and methods

**Fig 1:**
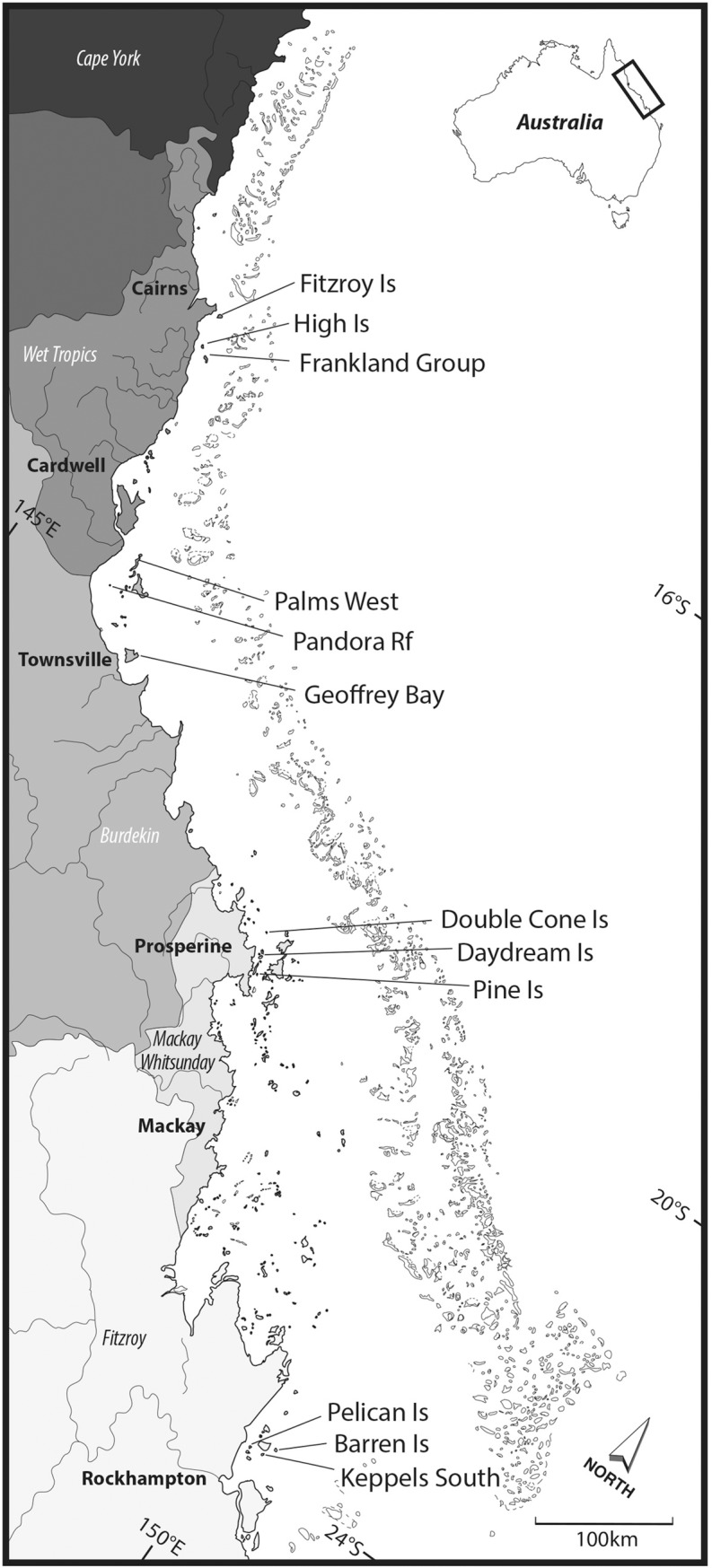
Sampling locations. From north to south, sampling locations are grouped into four regions (shading darkest to lightest): Wet Tropics, Burdekin, Mackay Whitsunday, and Fitzroy. Within each Region, three sampling locations lie along water quality gradients away from the coast and regionally important rivers.

Coral settlement was recorded annually on twelve reefs between 2006 and 2012. Three reefs were located in each of four catchment regions (Wet Tropics, Burdekin, Mackay Whitsunday, and Fitzroy regions, Fig 1). At each reef, two sites, separated by at least 250m, were selected. In the Burdekin Region, sites at Orpheus Island and Pelorus Island were combined as reef ‘Palms West’; in the Fitzroy Region, sites at Humpy Island and Halfway Island were combined as reef ‘Keppels South’.

Within each site, five permanently marked 20m long transects followed depth contours at both 2m and 5m below lowest astronomical tide datum. For this study, on the 5m contour at each site, 18 unglazed terracotta tiles (115 x 115 x 11mm) were deployed in three loose clusters of six tiles, separated by approximately 50m. Tiles were fixed individually to stainless steel base-plates that were attached to reef substrate with masonry plugs or zip ties [40], or mounted on a stainless steel rod over unconsolidated silt, sand or rubble at sites where the substrate condition precluded use of base-plates.

To confirm the timing of spawning across regions, colonies of *Acropora* species common to the study sites and bordering the transects, were haphazardly inspected for egg presence and pigmentation [41] during the spawning season of 2006–2007. Up to five branches per colony were broken and visually inspected. Once eggs were observed no further branches were inspected on a colony. The results of this egg inspection are presented in Table A in S1 Appendix, a separate document of supporting information.

Settlement tiles were deployed each year to coincide with expected peaks in coral spawning at inshore reefs (S1 Table). In 2006, 2007, and 2008 dual deployments were timed to capture settlement of larvae spawned following full moons in October and November (1^st^ deployment), and larvae spawned following full moons in December and January (2^nd^ deployment). From 2009–2012 sampling was adjusted to a single deployment spanning the two most likely spawning moons; following full moons in late October–early November, and late November–early December [42–45]. Duel deployments were timed to ensure at least 10 days to condition tiles before settlement was expected, and left in place at least two weeks after spawning to allow settlement. In support of this strategy, [46] observed that terracotta tiles, conditioned for only five days and clean of visible encrusting biota, were “a highly preferred settlement substratum, possibly due to early biofilm development on the tile surface”. Competency for settlement was assumed to be within three to five days after spawning [9, 41–43]. Once the single deployment strategy was established, the conditioning period prior to the October spawning event among all reefs and all years (2009–2012) averaged 4 weeks (S1 Table). The conditioning period that encompassed full moons between October and December averaged 9 weeks, with an average deployment time of 11 weeks. This was enough post-settlement time for Acroporidae spat to lay down a recognisable skeletal structure that would enable family-level identification [47]. Unlike most coral settlement/recruitment studies, this research was logistically constrained by the large geographic spread of the study area (see also [48]) and weather conditions that hindered deployment and retrieval, causing the variable conditioning periods observed between regions and between years (S1 Table). As an example, during the 2010–2011 wet season flood of the Fitzroy River, retrieval of settlement tiles at Pelican Island was delayed due to adverse conditions, leaving the tiles deployed for a maximum of 24 weeks.

Upon collection, tiles were stacked onto frames that ensured surfaces were kept apart and coral spat were not damaged or dislodged. Tiles were then bleached in a weak (0.3%) solution of sodium hypochlorite for 24 hours to remove all live tissue, rinsed in freshwater, and dried. Identification and enumeration of bleached spat skeletons on each surface was made with the aid of a stereo dissecting microscope. Taxonomic resolution was limited to the following categories: Acroporidae (not *Isopora*), *Isopora*, Fungiidae, Poritidae, Pocilloporidae and ‘other families‘, following [47] as the primary guide to identification.

Statistical analyses were constrained to the family Acroporidae, which, on average, represented 84% of observed settled corals. The remaining settlement included Poritidae 5%, Pocilloporidae 5%, with the remaining taxa (including Isopora and Fungiidae) rare or unidentified. Prior to analysis, the mean settlement per tile was estimated for each reef and year. Where dual deployments were used (2006, 2007, 2008) a single mean was estimated as the sum of the mean settlement for the two deployments.

Covariates in analyses included:

- Percent cover of adult Acroporidae, macroalgae, and soft coral, were estimated from five 20m long transects at each of 2m and 5m depths at the tile deployment sites. Along each transect digital photos were taken at 50cm intervals from which 32 photos were randomly selected. During photo analysis, five fixed points were superimposed on each photo-frame and the benthos under each point identified (adapted from [49]). The proportion of points scored for each of our target covariates was used as an estimate of their benthic cover. For data analysis, the cover of each covariate was taken as the average from both 2m and 5m depths as we consider the potential source of brood-stock (Acroporidae) and the influence of potential inhibiting allelochemicals (macroalgae, soft corals) to encompass both 2m and 5m depths. Analysis categories that scored for Acroporidae juveniles (<10cm size) were excluded and the Acroporidae recorded were considered as adult. The extent of their benthic cover was used as a relative estimate of potential local brood-stock. Benthic sampling occurred in the winter preceding the spring/early summer tile deployments following the MMP field schedule.
- The percent cover of CCA on tiles at the time of collection was visually estimated as a proportion of each tile surface, and averaged across tiles. While we cannot know what the CCA cover was at the time of coral settlement, cover at collection was used to determine relative differences between tiles across deployments.
- Water turbidity during the probable pelagic larval stage and at fertilization was estimated from in situ sensor measurements. At each reef, WET Labs ECO FLNTUSB (combination fluorometer and turbidity sensors) were co-located with the first cluster of coral settlement tiles. Turbidity (in nephelometric turbidity units, NTU) was recorded at 10 minute intervals. Mean turbidity during the pelagic larval stage was calculated from the 12 days following each of the two most likely spawning days defined as 5 days following either late October or early November, and late November or early December full moons. Mean turbidity at time of fertilisation was calculated from the 3 days following each of the two most likely spawning dates as defined above. Due to occasional sensor failure, a small number of turbidity records were missing. In these cases, estimated turbidity values were derived from linear models fitted separately for each location that parameterised turbidity as a function of daily observations of wave height and tidal magnitude, following methods in [50]. Where turbidity values are discussed with comparison to other studies that measure total suspended solids (TSS), our turbidity estimates are converted to TSS in mgL^-1^ using the conversion TSS = NTU*1.33, an equation based on a comparison between direct water samples (TSS) and instrument turbidity readings (NTU) [51].
- The rate of sedimentation was estimated as the daily mean dry weight per cm^2^ of sediment captured in sediment traps. Three sediment traps were deployed at each reef, 25 m apart along the 5 m transects. A sediment trap was a 400mm long cylinder of 100mm diameter and was attached to a metal stake to raise it from the substrate. Sediment traps were left in place for 30–160 days between December 2010 and June 2012. Sediment samples were retrieved and wet-sieved to separate the <63 μm fraction (clay-silt, [52]). Sieved samples were washed with freshwater to remove salt content before being oven dried at 75°C and weighed to calculate the average daily deposition of clay and silt sized particles. Given the limited period of sediment trap deployments in this study, sedimentation data were used for spatial analysis only.
- Annual river discharge data (October to September) was provided by the Queensland Department of Natural Resources, Mines and Energy [53]. For analysis, annual discharge from each region’s major rivers was expressed as proportional to the long-term median (1986–2016) (Table 1). Flows were corrected for ungauged area of catchments [32,54].

**Table 1:** Annual freshwater discharge over the study period for the four GBR catchment regions influencing the study reefs.

Region	LT Median (ML)	2006	2007	2008	2009	2010	2011	2012
Wet Tropics	20,978,805	1.3	1.1	1.1	1.4	1.1	2.4	1.2
Burdekin	5,976,064	0.6	2.3	5.6	6.0	1.9	7.3	3.4
Mackay Whitsunday	3,514,304	0.4	1.4	2.3	1.5	2.7	4.9	2.4
Fitzroy Basin	3,568,539	0.3	0.4	4.3	0.9	4.2	12.3	2.8

Flows expressed as a proportion of the long-term median (megalitres ML), and were corrected for ungauged area of catchments.

The degree to which spatial (among reef) and temporal (within reef) variability in Acroporidae settlement could be attributed to explanatory variables was analysed separately. In both cases the response variable was the mean number of spat per tile. Temporal variation in settlement was investigated using Generalised Additive Mixed Models (GAMM) fitted using the mgcv package [55, 56] within the R statistical and graphical environment [57]. Candidate models comprising combinations of covariates (including null, intercept only models) were fitted via maximum likelihood and compared via Akaike's Information Criterion corrected for small sample sizes (AICc).

Models were validated by exploring patterns of the residuals. The base null model included reefs as a random effect to account for spatial variation, pseudo-replication and temporal autocorrelation arising from multiple and repeated observations from the same reefs. The null model also included a random effect representing the change in sampling design from double to single tile deployments as preliminary investigations provided inferential support for this effect. Given the relatively small sample size, the inclusion of two random factors to account for repeated measures, and the variability introduced because of the sampling design, separate models were compared to the null model to assess relationships. These separate models included covariates of: reef-level cover of adult Acroporidae (as an estimate of brood-stock); cover of macroalgae and cover of soft coral (as potential recruitment deterrents); turbidity (as a measure of environmental conditions); and CCA cover on tiles (as a measure of settlement substrate condition). To identify natural trends in explanatory variables they were initially incorporated as beta splines and simpler linear models selected where no curvature in response was indicated. To improve normality and reduce heteroskedasticity, the response was logarithmically transformed. Given the substantial variation between reefs, the explanatory variables (with the exception of sampling years) were log transformed to focus on proportional differences in covariates within reefs. Models selected based on AICc (i.e. models with an AICc at least two units below that of the null model) were refitted via Restricted Maximum Likelihood, the proportion of variability in settlement explained by the covariates of interest estimated and predicted partial effects plots produced.

Differences in mean settlement among reefs over all sampling years were investigated by individual linear models that regressed the log transformed mean spat per tile at each reef against the mean levels of each explanatory variable. Single explanatory variable models were used due to the small number (12) of response observations.

Permission for the field component of this study was given by the regulatory authority for the Great Barrier Reef; the Great Barrier Reef Marine Park Authority.

## Results

The estimation of timing of Acroporidae spawning, and the optimisation of deployment duration of settlement tiles is detailed in supporting information S1 Appendix. Most eggs were developed towards pre-spawn condition by November-December (Table A in S1 Appendix), and that the optimal deployment period for tile conditioning and larval settlement was from October to December (Table B in S1 Appendix). During the study a total of 4104 tiles were deployed, with the loss of only 21 during storm events.

### Relationships between settlement and environmental variables

**Fig 2:**
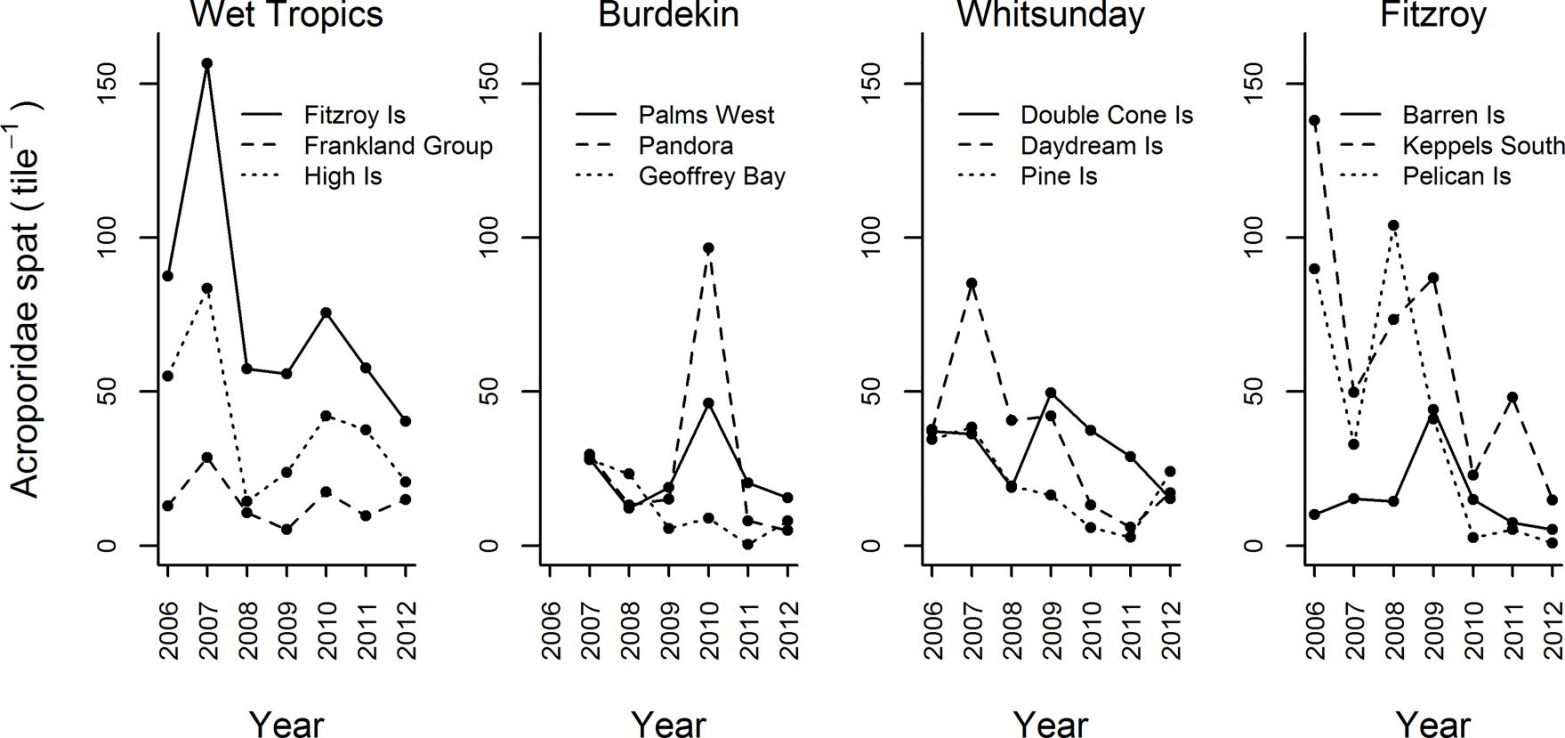
Time series of settlement of Acroporidae spat in four regions. The position of each sampling location along the water quality gradient is identified by line style: dotted line—most inshore reef; dashed line—intermediate; solid line—most offshore reef. A table of annual means and standard deviations for all reefs is given in supplementary information S2 Table.

Over the seven year study period a total of 116,364 spat were recorded. Of these, 84% were Acroporidae, 5% Poritidae, 5% Pocilloporidae, and 6% were combined Isoporidae, Fungiidae, and undifferentiated taxa (0.23%, 0.19%, 5.63% respectively). Across all reefs and years, Acroporidae spat settled at an average density of 33.55 ± 31.01 (mean ± SD) spat per tile. However, settlement was highly variable among reefs and between years with distinct pulses of settlement observed in each region: Wet Tropics 2007, Burdekin 2010, Mackay Whitsunday 2007, Fitzroy 2008, 2009 (Fig 2). For annual reef-level counts of identified taxa, and mean and standard deviations of Acroporidae, see supplementary information S2 Table.

**Fig 3:**
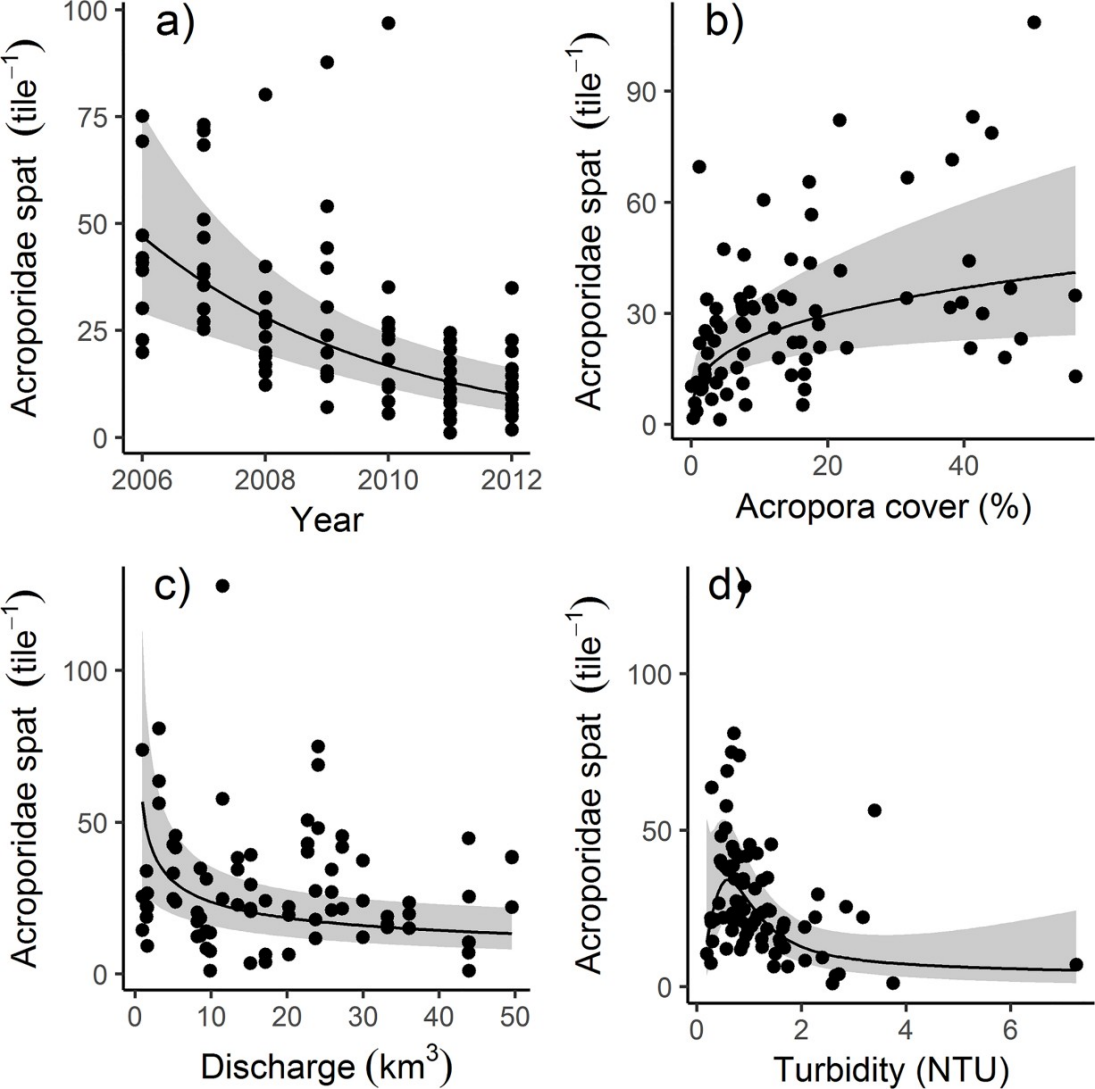
**Partial effects plots of explanatory variables to Acroporidae settlement for**; **a**: Sampling years, **b**: Acroporidae cover, **c**: River discharge, and **d**: Turbidity. Shaded areas represent 95% confidence intervals of the predicted relationship.

**Fig 4:**
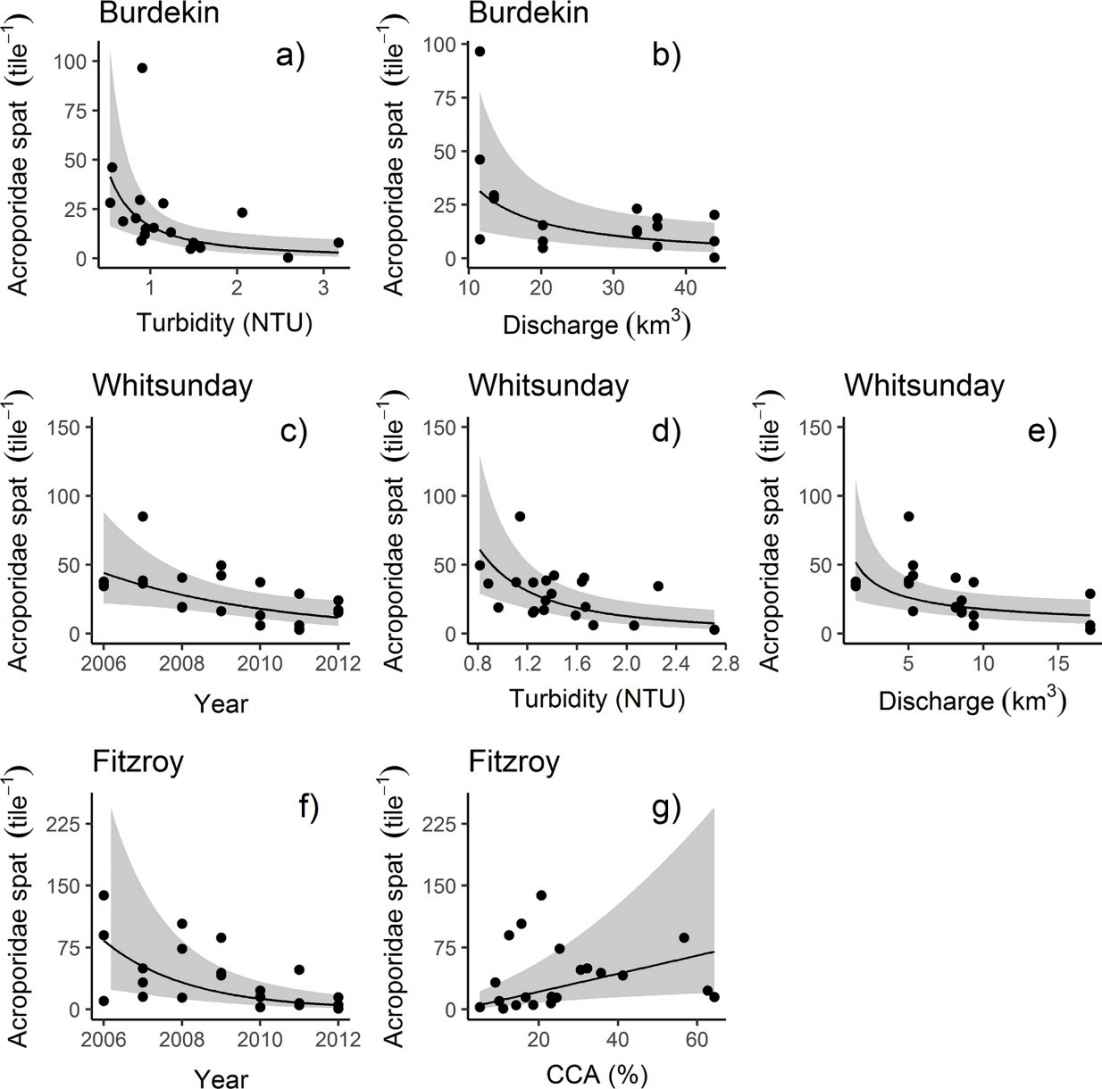
Partial effects plots of Acroporidae settlement. Showing fitted relationships with regionally significant covariates as determined by model selection (Table 3). For Burdekin **a**: Turbidity (NTU), **b**: River discharge (km^3^). For Mackay Whitsunday **c**: Sampling years, **d**: Turbidity (NTU), **e**: River discharge (km3). For Fitzroy **f**: Sampling years, **g**: Coralline algae cover (%). Shaded areas represent 95% confidence intervals of the predicted relationship.

Of the covariates analysed, Year, Acroporidae cover, River discharge, and Turbidity variously corresponded to temporal variation in settlement (Table 2, Fig 3). Settlement declined over the duration of the study (Fig 3A) with Year explaining 21.5% of the observed variation (Table 2). Among regions (Fig 4), overall declines in settlement were primarily driven by declines in the Mackay Whitsunday and Fitzroy regions (Fig 4C and 4F, Table 3), but were also influenced by the very high settlement recorded in the Wet Tropics in 2007 (Fig 2). Notably, for eight of the nine reefs outside the Wet Tropics Region, the lowest settlement occurred during the last years of this study; 2011 and 2012 (Fig 2).

**Table 2:** Model selection using Akaike’s information criterion (AICc) across all regions.

Explanatory variable	Random Effects	AICc	Fixed effects R^2^	Model R^2^
Null	Reef	240.5	na	0.216
Method	Reef	**228.3**	0.124	0.369
Null	Reef + Method	233.7	na	0.437
Year	Reef + Method	**217.3**	0.215	0.543
Acroporidae cover	Reef + Method	**226.7**	0.154	0.509
River discharge	Reef + Method	**228.6**	0.120	0.534
Turbidity (pelagic)	Reef + Method	**229.0**	0.082	0.483
Turbidity (fertilisation)	Reef + Method	232.0		
Soft coral cover	Reef + Method	233.6		
Crustose coralline algae cover	Reef + Method	236.0		
Macroalgae cover	Reef + Method	237.1		

In each case the null models include random effects to which models lower in the table are compared. For supported models (bold AICc), R^2^ values for the explanatory variable and full model are provided.

**Table 3:** Model selection using Akaike’s information criterion (AICc) within regions.

Explanatory variable	Wet Tropics	Burdekin	Mackay Whitsunday	Fitzroy
Null	50.9	65.6	58.3	76.4
Acroporidae cover	52.9	69.5	58.7	75.5
Turbidity (pelagic)	52.3	**61.0** **(0.380)**	**51.1** **(0.351)**	79.9
River discharge	53.4	**62.1** **(0.253)**	**54.9 (0.250)**	77.8
Year	50.8	66.5	**55.4** **(0.296)**	**69.2** **(0.408)**
Macroalgae cover	51.1	69.4	60.7	79.9
Soft coral cover	53.8	68.9	57.5	79.2
Crustose coralline algae cover (CCA)	53.2	69.4	61.6	**72.1** **(0.211)**

In each case the null model included random effects for reef and method. For supported models (in bold) R^2^ values for the explanatory variable are included in brackets.

**Fig 5:**
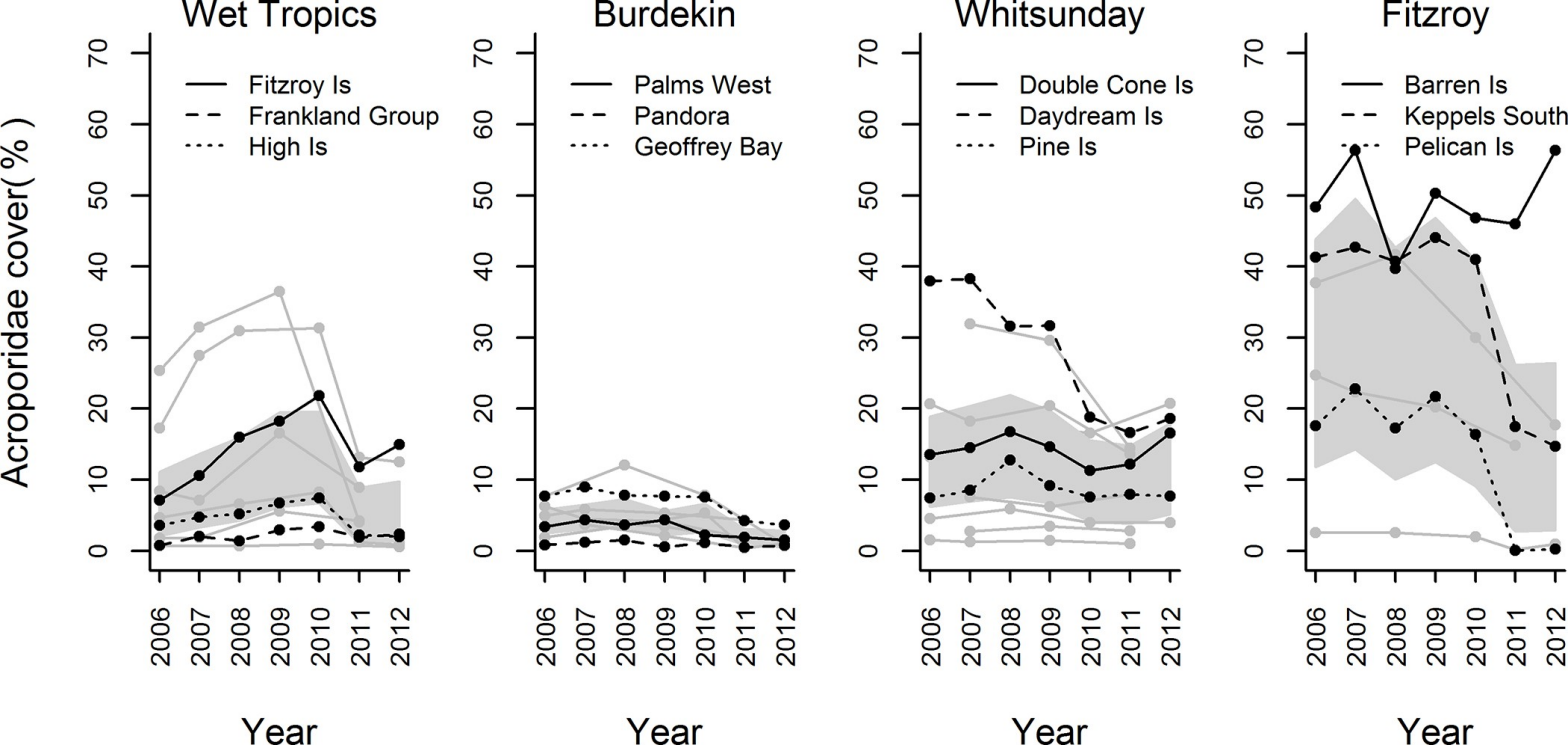
Time-series of adult Acroporidae cover in four regions. The position of each sampling location along the water quality gradient is identified by line style: dotted line—most inshore reef; dashed line—intermediate; solid line—most offshore reef. Grey lines represent Acroporidae cover at other, nearby, reefs in each region [58]. Shaded areas represent the 95% confidence interval around mean trend as estimated from generalised mixed effects models in which all reefs (including nearby reefs) are incorporated as random effects.

**Fig 6:**
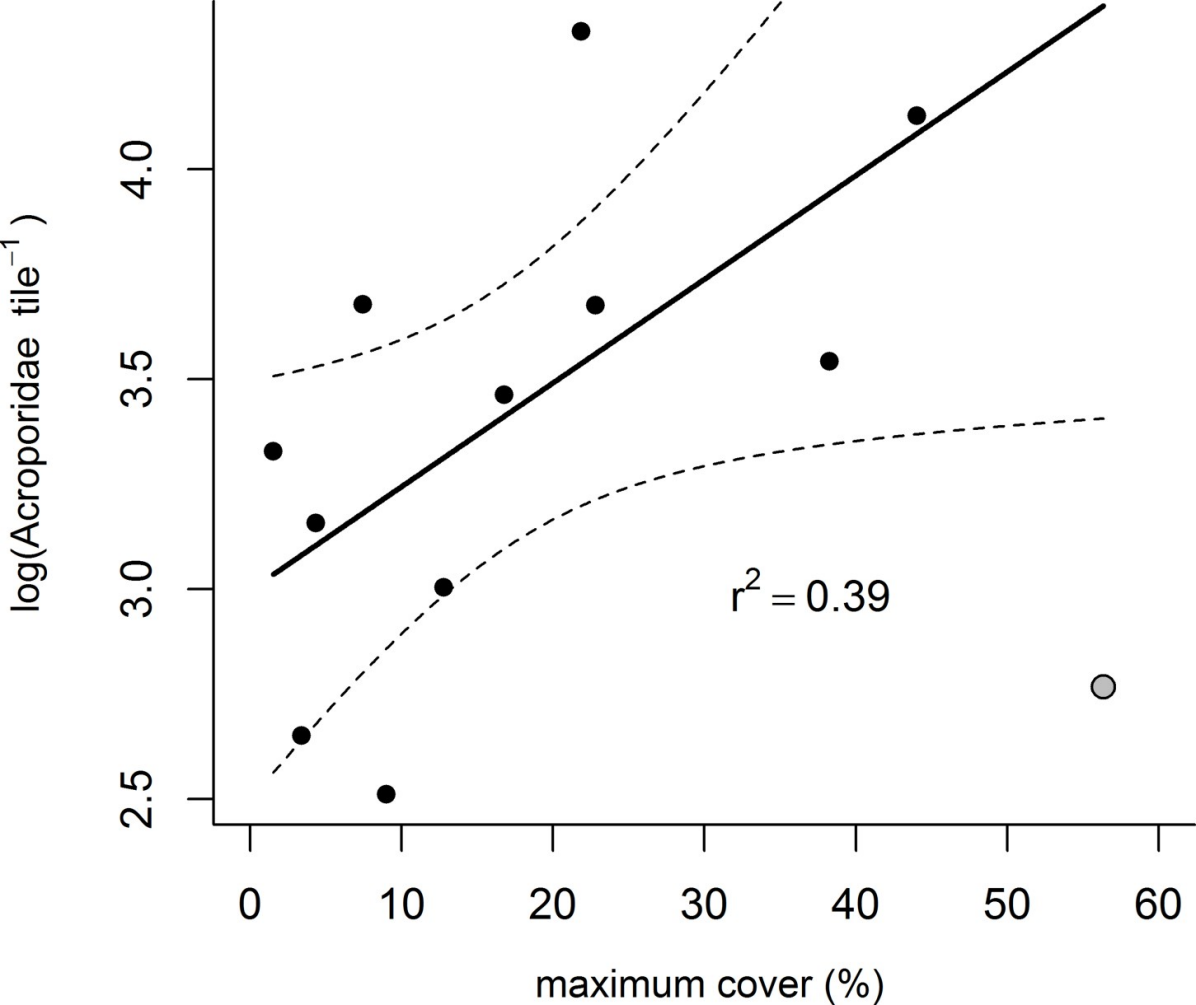
Relationship between settlement and the maximum cover of adult Acroporidae. Fitted line was derived with the exclusion of Barren Island (grey symbol).

Differences in cover of adult Acroporidae explained only 15% of the overall variation in settlement over time (Table 2), although cover did not strongly correspond to settlement in any single region (Table 3). The positive relationship between settlement and Acroporidae cover is highly variable, particularly at moderate and high levels of settlement (Fig 3B). Time-series data of Acroporidae cover shows a close relationship among most reefs (Fig 5); in each region declines in cover at survey reefs were generally evident at other reefs in the region indicatinig regional decline in brood-stock (annual means and standard deviations for all reefs are given in S3 Table). The most likely cause of declines in Acroporidae cover in the Wet Tropics and Burdekin Regions in 2011 was Tropical Cyclone Yasi. Flooding associated with a southward moving monsoon trough was responsible for the marked declines in Acroporidae cover at 2m depths in 2011 in the Fitzroy Region. Cylone Ului (2010) had a variable impact on Acroporidae cover in the Whitsunday Region (Fig 5). Adult Acroporidae cover was also the only covariate that explained spatial variability in settlement, with the correspondance between reef level settlement and Acroporidae cover marginally higher when maximum Acroporidae cover was considered (R-square 0.39, P = 0.04, AICc = 21.4, Fig 6) compared to the mean cover of Acroporidae over the 7 years of the study (R-square 0.35, p = 0.056, AICc 22.1). However, the relationship between Acroporidae cover and reef level settlement was only evident when Barren Island was removed on the basis of that site being a statistical outlier (Cooks distance>1, Fig 6). Annual means and standard deviations for all reefs are given in S3 Table

Evidence of water quality influencing settlement was observed both as a negative relationship between discharge from local rivers in the wet season preceding settlement (Fig 3C), and turbidity (NTU) during the pelagic larval phase (Fig 3D). The overall variation in settlement explained by water quality metrics was low at just 12% for river discharge and 8% for turbidity. In both cases relationships were evident in both the Burdekin (Fig 4A and 4B) and Mackay Whitsunday (Fig 4D and 4E) regions (Table 3). The nonlinear response of settlement to turbidity (Fig 3D) was initially influenced by the consistent low turbidity / low settlement at Barren in the Fitzroy Region which effectively precludes a relationship between turbidity and settlement in that region.

Neither changes in macroalgae cover during the preceding winter period nor soft coral cover improved the fit of models across all regions or within individual regions (Tables 2 and 3). However, as our macroalgae data was obtained preceding the spring/early summer tile deployments we do acknowledge that macroalgae estimates represent minimums for the season, and that settlement numbers may have been compromised by planulae dissuaded by the summer growth of macroalgae ([21–23]). Inclusion of CCA cover on the tiles at time of retrieval as a covariate did not improve model fits, except for the Fitzroy region (Table 3, S4 Table, S5 Table, S6 Table) where the positive relationship between settlement and CCA reflected relatively very low settlement that occurred on several occasions when CCA cover was below 20% (Fig 4G).

## Discussion

The inshore reefs of the GBR present a particularly challenging environment for corals, with pressures associated with poor water quality adding to the acute impacts resulting from thermal stress, cyclones, and predation by *Acanthaster* cf *solaris* experienced by reefs in general [4, 9, 50]. With projections for an escalation of climate related pressures [59], it is important to improve our understanding of the processes that may limit the ability of coral communities to recover from inevitable disturbance events. Notable in our time-series of Acroporidae settlement was high variability at both spatial and temporal scales, which conforms to previous research of settlement and recruitment patterns on the GBR [60–62]. We discuss this variability both in terms of the factors limiting the supply of larvae to reefs that potentially limit recovery, and the caution required when interpreting settlement patterns observed in studies of limited spatial or temporal replication.

### Adult Acroporidae cover

Acroporidae settlement was weakly associated with both spatial and temporal differences in the cover of Acroporidae, highlighting the potential links between the local brood-stock, but also the likely importance of additional factors, such as the hydrodynamic connectivity between populations [33, 34, 63–67] and the replenishment of coral communities at some reefs. Spatially, the weak positive relationship between settlement and brood-stock indicates locally retained larvae may have been a source of replenishment at some reefs [63]. However, this result was only supported with the exclusion of Barren Island, the site with the highest cover of Acroporidae and consistently low settlement, suggesting larval retention was limited. The coral community into which the tiles at Barren Island were deployed consisted almost entirely of the staghorn species *Acropora muricata* and *A*. *intermedia*, thickets of which consistently harbour very low densities of juvenile corals (authors’ pers. obs.). This low density of juveniles was possibly due to the small size of this reef and potentially higher flow rate, given it’s more exposed geographical position, promoting advection rather than retention of larvae [68].

Temporal variation in settlement within individual reefs could be typified as being either consistently low, when both reef level and regional brood-stock were low as a result of regional level reductions in coral cover, or highly variable when regional cover was higher. From this result it appears that the regional losses of brood-stock associated with Cyclone Ului in the Whitsunday Region, Cyclone Yasi in the Wet Tropics and Burdekin regions, and severe flooding in the Fitzroy Region, were sufficient to substantially limit larval supply in subsequent years. Conversely, high variability in settlement prior to these disturbances, when brood-stocks were relatively stable, implies either variable supply of competent larvae or variable sampling success of the tiles. The limitations affecting the sampling success of the tiles are discussed in a later section. Variable supply of competent larvae can occur as a result of variable connectivity to brood-stock or variation in the fecundity of those brood-stock [34], [69], and variable rates of larval survival though to settlement. Fecundity of corals can be influenced by environmental stressors including: reduced light levels [17], high nutrient availability [70], thermal stress [19, 71] and the resulting partial mortality [72]). As our study did not include annual assessments of fecundity, current dynamics, or survivorship of larvae, we cannot separately identify the magnitude of their influence on patterns of coral settlement.

### River discharge

Most of the freshwater discharge to the GBR lagoon (January to March) occurs during the Austral summer monsoons which increases the turbidity of inshore waters for periods of several weeks to months [73, 74]. Significant flood events very early in the wet season, i.e. before mass coral spawning, are rare (see hydrographs in [75]). Hence, effects of river discharge on recruitment processes are more likely through responses of the brood-stock and substratum condition to pressures from the preceding wet season. Over the duration of this study the highest discharges in each region occurred in early 2011; in the aftermath of Cyclone Yasi and monsoonal downpours in the Fitzroy Region, leading to widespread exposure to flood waters. The co-occurrence of high discharge and loss of coral cover following Cyclone Yasi confounds interpretation of any sub-lethal influences of discharge that may have reduced fecundity during the next spawning event. However, the onset of heavy rainfall early in the 2010/2011 wet season in the Fitzroy region that coincided with November 2010 spawning, raises the prospect that, in this instance, settlement was low due to reduced fertilisation as a result of gamete exposure to hyposalinity [76]. Significantly, the flood event during December 2010 to March 2011 caused 100% mortality among the Acroporidae brood-stock at Pelican Island effectively restricting replenishment to inter-reefal dispersal in later years. Notably, within the broader MMP there is a negative relationship between changes in a multi-criteria index of coral community condition and discharge from local catchments that is consistent with runoff-induced stress to the benthic community [77] and the observed negative relationships between discharge and subsequent settlement success.

### Turbidity

Turbidity showed no effect on settlement, with only a weak association between turbidity levels during the pelagic phase and spat numbers (Table 2). While turbidity levels are acknowledged to be critical during the early lifecycle phases, our in situ logger readings over this study period show turbidity levels during the pelagic larval stage were consistently below those levels found to affect Acroporidae larvae under experimental conditions (see NTU plots in [75]). The highest average turbidity level recorded in this study for the pelagic phase was 7.07 NTU (TSS 9.4mgL^-1^, Pelican 2011), with the majority of records below 3.75 NTU (TSS 5mgL^-1^, Fig 3D). By contrast, experimental turbidity levels were in the range of 37.5 – 75NTU (TSS 50–100 mgL^-1^ [29, 78]), and as high as 173NTU (TSS 230 mgL^-1^ [79]), before causing a reduction in coral fertilization and influencing the settlement and metamorphosis process. Further, the comprehensive study by [27] showed reductions in fertilisation and settlement began only when turbidity levels of 22.5 and 75 NTU (TSS 30mgL^-1^ and 100 mgL^-1^) were additional to an another stressor, such as higher temperature (32°C), or higher organic nutrient concentrations (0.6 mg organic carbon L^−1^). This strongly suggests that naturally occurring turbidity levels were not the primary factor influencing the negative relationship in our results.

The results of this study support earlier research showing the peak coral spawning period in the inshore waters of the GBR occurs between late October and early December [41, 42, 80]. The timing of spawning thus precludes exposure of larval corals to the precipitous rise in turbidity associated with river discharge that typically occurs between January and March [75], depending on the particular catchment and year. Given the disparity between recorded turbidity levels and those that experimentally affect the pelagic larval phase, we conclude that recorded turbidity levels may be a proxy for another limiting process, principally connectivity to brood-stock. Increased turbidity over the larval period is primarily the result of wind-driven resuspension ([50], reviewed in [81]). Variability in resuspension can be interpreted as variability in surface currents that may, in turn, indicate variability in dispersal patterns of coral larvae [82]. Indeed, the settlement pulses to reefs with very low cover of adult colonies in our study evidences the sporadic connectivity to brood-stock beyond the immediate vicinity of the sites [37]. For example [33] and [83] reported highly variable settlement at small spatial scales, with relatively high spat densities at sites where eddies were predicted to form and persist, and suggested local hydrological conditions within single reefs, at scales of a few kilometres, were driving settlement patterns. Babcock [65] reported the possible influence of strong winds on spawn slicks that caused an interruption in the sequence of settlement patterns at a reef.

### Influence of surface quality on settlement

Our goal was to estimate the number of Acroporidae spat capable of settling each year at nominated inshore reefs during the peak two-moon spawning / settlement cycle. Consequently, we chose to deploy settlement tiles to condition for the minimum time that would allow the development of a biological surface conducive to settlement in time for the first lunar cycle. Importantly, we wished to reduce the risk of the corresponding development of competitive invertebrate mats that could overgrow settled corals [84] if tiles remained deployed much later than the second-moon cycle. Both [46] and [85] confirmed that tiles deployed a short time (≥ 5 days) ahead of the first expected settlement would be conditioned enough to attract planulae to settle, actively cued by the presence of surface microbial biofilms, and in advance of the slower developing CCA cover. This was further supported by [86] who reported that, under experimental conditions, a microbial biofilm surface developed over only two weeks was equally effective as one developed over two months, with similar high settlement rates of 72%. We believe our deployment strategy, spanning full moon spawning / settlement cycles between October and December, and tile conditioning for an average of 24 days (range 11–42) prior to the first expected settlement phase, allowed for the adequate development of microbial films and crusts of CCA. Importantly, our findings show that, in this study, variability in CCA cover was not associated with the length of conditioning period (S4 Table), and had little effect on the numbers of settling Acroporidae.

An important consideration when deploying tiles for our settlement study was that, as we were not trying to emulate the surrounding benthos, the limited conditioning could introduce a bias due to the sampling efficiency of a relatively ‘fresh’ surface free from the more mature benthic layers in the surrounding habitat. Successional changes in microbial films and CCA species can alter the chemical cues and cause a reduction in attractiveness of the substrate over time [87]. As coral planulae are known to ‘test’ the substrate prior to settlement [88, 89] the relative attractiveness of a lightly conditioned tile compared to the surrounding substrate may vary the proportion of larvae settling to tiles at small spatial scales [33]. We suggest that at reefs where the natural substrate is unattractive to searching larvae, the tiles may offer a preferable substrate, confounding our analyses comparing settlement to factors of the benthic community known to influence settlement: the presence of CCA on tiles [90–92]; local cover of macroalgae [23, 93] and soft corals [94, 95], or rate of sedimentation [9, 25, 26, 89, 93, 96].

At the scale of individual tiles the sensitivity of larvae to variation in conditions is well documented, with settlement preferences shifting from the underside of tiles in shallow, well-lit water, to narrow vertical surfaces, then to the upper tile surface in deeper water or in poor light availability [28, 33, 60, 88, 97]. In our study 51% of Acroporidae settled to the narrow vertical sides of tiles comprising just 10% of the surface area (S5 Table), a preference interpreted by others as reducing post-settlement mortality from grazing, algal growth and sedimentation while still maintaining exposure to light [28, 98–100]. The highest rates of sedimentation in our study occurred at the Mackay Whitsunday region reefs. Although not quantified, settlement tiles retrieved from these sites had regularly accumulated a thick layer of sediment on upper surfaces beneath which there was little or no growth of any type, demonstrating that highly sediment-laden substrata precludes settlement of a range of organisms. Across all survey years, 75% of Acroporidae settlement at our Mackay Whitsunday reefs occurred on the sides, or undersides of our tiles. This result strongly suggests there would be limited settlement on sediment-laden surrounding substrate.

### Improving settlement tiles as a monitoring tool

For the study of coral settlement there are few alternatives to the use of standardised settlement surfaces. High variability at both spatial and temporal scales is common with such studies, necessitating deployment of a high number of replicates ([48], [36], this study). However, the deployment of coral settlement tiles is labour intensive and weather dependent, resulting in logistical constraints that, at the scale of our study, limited our control of factors contributing to settlement success. Of notable concern was the inconsistency of the tile conditioning period in preparation for a single deployment over two spawning dates, across all reefs and all years (S1 Table, S4 Table). With reefs spread over more than 875km it was necessary to take advantage of available weather windows in the month pre-spawning to ensure tiles were in place at all reefs prior to the first expected spawning date.

Ideally, for a more rigorous study of inter-annual coral settlement, local egg colour would be checked to ascertain spawning likelihood prior to each spawning period [63], and tiles would be deployed accordingly. To limit overlap between spawning events following consecutive full moons, we suggest tiles preconditioned in filtered, larvae-free, water be deployed at the time of collection of the previous month’s tiles to limit exposure to first-period larvae [65, 86]. While this initial conditioning would not reflect each reef’s microbial signature, it would allow a degree of standardisation to be imposed on the tile surfaces, a consideration recognised by the current study. Necessary improvements to future monitoring of coral settlement would require regional-scale surveys of adult cover and fecundity [34], more uniformly conditioned tiles afforded by deployments targeted to individual spawning events and, crucially, incorporation of both hydrodynamic and genetic modelling to better identify potential sources of larvae from self-seeding or dispersed sources [82, 101–103].

Alternatively, changes in the density and taxonomic richness of juvenile corals (≤5cm diameter, [58]), measured using belt transects, are likely to provide a more reliable indication of coral community health [104] as juveniles are sensitive to changes in the local habitat (e.g. sedimentation, water quality, reviewed [5]) and represent a life-cycle stage that has passed through the bottle-neck of high recruitment mortality [105–106].

## Conclusion

We have identified the main factors that we believe limit the utility of settlement tiles as a long-term monitoring method. We stress that these issues are unlikely to be specific to our study, and that the variability we observed should be considered when making inference from other studies reporting spatial or temporal patterns in settlement. We suggest that monitoring fecundity may provide a more direct assessment of environmental conditions limiting reproductive output of populations, while monitoring the density of juvenile corals is a better indicator of the success of the entire recruitment process. In combination with hydrodynamic modelling, this approach would include the net influences on replenishment due to reduced fecundity, variable dispersal, the condition of settlement surfaces on the reefs, and early post-settlement mortality [105, 107].

This study demonstrates abundant, although variable, settlement of Acroporidae larvae on inshore reefs of the Great Barrier Reef. The study also placed the influence of candidate covariates within the context of the water quality regimes of the inshore environment. Our observation of consistently low coral larvae settlement following the regional loss of Acroporidae cover due to the impact of cyclones and flooding indicates the density dependence of settlement. The variability around this relationship likely to reflect the additional influence of the hydrodynamic processes linking brood-stock populations to a particular site, and variability in fecundity of that brood-stock [34].

The back-to-back bleaching events of 2016 and 2017 underline the vulnerability of the GBR to widespread loss of brood-stock at scales large enough to be of concern for the health of the GBR [108]. Modelling by [82] highlights that the resilience of the GBR relies on the flow of larvae from highly connected ‘robust source reefs’ that have a lower risk of experiencing severe disturbance (cyclones, bleaching, *Acanthaste*r cf *solaris* outbreaks). According to [109] a rise in ocean temperature around reefs will result in decreased inter-reef connectivity and increased self-seeding as more coral larvae attain competence before leaving the natal reef complex. Inshore reefs may be particularly susceptible to recruitment-cycle limitations as they are subject to the combined effects of intensifying global stressors, additional stressors of the coastal environment [5] and potential isolation from brood-stock refugia.

## Supporting information

S1 Appendix Determining the time of spawning and the deployment duration of settlement tiles. (DOCX)

S1 Table Tile deployment periods at each study reef. The date of the Full Moons during the main spawning season was used to estimate the number of tile conditioning days (C) before competency of settlement of Acroporidae larvae (assumed to be 10 days post-full moon [42]), with the total number of tile deployment days (D) before collection. For years with two tile deployments (2006–2008), the dotted line indicates the limits for conditioning and deployment, when the first set of tiles were retrieved and replaced with a second set of clean tiles.
(TIF)

S2 Table Family groups of spat reported for each reef and each year of study. For each reef the annual totals for Acroporidae, Poritidae, Pocilloporidae, Isopora, Fungiidae, and undifferentiated spat are listed. For each taxonomic group, the total across the study period and the percentage representation is given. In addition, the mean, standard deviation, and standard error are calculated for settled Acroporidae for each reef and each year. In years with dual deployments (2006–2008), the reef mean was estimated as the sum of the mean settlement for the two deployments, averaged over the two sites.
(XLSX)

S3 Table Average cover of adult Acroparidae (%) reported for each reef and each year of study. **For each reef the annual estimate of average Acroporidae cover is given.** In addition, the mean, standard deviation and standard error are given. The principle reefs studied are in bold. The other locations on the list are neighboring reefs where monitoring is conducted bienially.
(XLSX)

S4 Table Tile conditioning in days and the average % CCA cover on recovered tiles. Where C = the maximum conditioning period from deployment to +10 days post 2^nd^ (or 3rd if applicable) moon during spawning season, and cover of CCA (%) is an indicator of the level of exposure of coral larvae to CCA among reefs and years.
(DOCX)

S5 Table Distribution of Acroporidae spat on settlement tile surfaces. Total Acroporidae per surface (Top, Bottom, Edge), and the proportion of Acroporidae spat on each surface for each year at each reef.
(XLSX)

S6 Table Distribution of crustose coralline algae (CCA) on settlement tile surfaces. Mean cover of CCA (%), from estimated CCA cover on each settlement surface (Top, Bottom, Edge) from each tile for each year at each reef.
(XLSX)
