# Supplementary material for: High spatio-temporal variability in Acroporidae settlement to inshore reefs of the Great Barrier Reef
Source: PLoS One. 2019 Jan 30;14(1):e0209771. doi: 10.1371/journal.pone.0209771 (PMC6353100; doi:10.1371/journal.pone.0209771)
Supplement: S1 Appendix — (DOCX) [file pone.0209771.s001.docx]

## The following supporting information accompanies the article:

**High spatio-temporal variability in Acroporidae settlement to inshore reefs of the Great Barrier Reef.**

Johnston Davidson^1*^, Angus Thompson^1^, Murray Logan^1^, Britta Schaffelke^1^

^1^Australian Institute of Marine Science, Townsville, Queensland 4810, Australia

^*^ Corresponding author: [j.davidson@aims.gov.au](mailto:j.davidson@aims.gov.au)

**S1 Appendix.**

**Timing of spawning of Acropora corals**

Progression of egg pigmentation in *Acropora* corals suggested that, for 13 species, spawning was split between early November 2006 and early December (Table A). In all regions, eggs observed prior to the full moon on the 7^th^ of October were un-pigmented indicating that spawning following this full moon was unlikely. The only exception was in the Fitzroy region where a very small proportion of colonies had pigmented eggs on the 6^th^ of September. The Fitzroy region was the only region where sampling was undertaken prior to the November 5^th^ full moon and a mixture of pigmented and unpigmented eggs were observed. Sampling prior to the December 5^th^ full moon in in all regions revealed a combination of colonies having pigmented eggs indicating imminent spawning, while the absence of eggs in others implied spawning following the preceding November moon.

**Table A. Egg presence and pigmentation over the 2006/2007 spawning season.** Column headings indicate sampling dates. Numbers in cells represent the number of colonies checked, letters denote state of oogenesis: ‘a’ eggs absent or not observed, ‘w’ immature (white) eggs, and ‘p’ mature (pigmented) eggs. Shading identifies when pigmented eggs were observed.

|  | Wet Tropics | | | | | Mackay Whitsunday | | | | | Fitzroy | | | |
| --- | --- | --- | --- | --- | --- | --- | --- | --- | --- | --- | --- | --- | --- | --- |
|  | 11^th^  Sep 06 | 28^th^  Nov 06 | | 29^th^  Jan 07 | | 5^th^  Oct 06 | 1^st^  Dec 06 | | 27^th^  Jan 07 | | 6^th^  Sep 06 | 30^th^  Oct 06 | 3^rd^  Dec06 | 24^th^  Jan 07 |
| Species spawning following 5^th^ November and/or 5^th^ December moons | | | | | | | | | | | | | | |
| *Acropora elseyi* | 9a,  5w | **7a,**  **4p** | | | 15a | 3w | 9a | | 10a | |  |  |  |  |
| *A. grandis* | 3w | **2a,**  **1p** | | | 5a | 2w |  | | 3a | |  |  |  |  |
| *A. hyacinthus* | 1a,  27w | **14a,**  **24p** | | | 41a | 5w |  | |  | | 1a,  9w | **7p** | **10a,**  **3p** | 31a |
| *A. intermedia* |  |  | | | 5a | 15w |  | |  | | **1a,**  **19w,**  **1p** | **13w,**  **10p** | **9a,**  **6p** | 34a |
| *A. latistella* | 10w | **17a,**  **2p** | | | 20a | 15w | **8a,**  **3p** | | 11a,  5w | |  |  |  | 3a |
| *A. millepora* | 6a,  58w | **14a,**  **81p** | | | 68a | 1a | **5a,**  **4p** | | 8a | | **5a,**  **32w,**  **2p** | **3a,**  **5w,**  **28p** | **28a,**  **1p** | 55a |
| *A. muricata* | 1a,  13w | 9a | | | 15a | 28w | **26a,**  **5p** | | 25a | | **2a,**  **66w,**  **1p** | **22w,**  **16p** | **25a,**  **12p** | 40a |
| *A. nasuta* | 45w | **3a,**  **50p** | | | 40a |  |  | |  | | 5a |  |  |  |
| *A. sarmentosa* |  | **3a,**  **5p** | | | 5a |  |  | |  | |  |  |  |  |
| *A. tenuis* | 44w | **15a,**  **74p** | | | 72a | 15w | **3a,**  **8p** | | 20a | | 21w | **3p** | **12a,**  **7p** | 13a |
| *A. valida* | 4a,  6w | 5a | | | 22a,  1w | 1a,  20w | 18a | | 22a | | 20w | **6w,**  **3p** | **11p** | 20a |
| *A. vaughani* |  |  | | |  | 5w | **2p** | | 5a | |  |  |  |  |
| *A. yongei* |  | 3a | | | 10a |  | **5p** | | 5a | |  |  | **3a,**  **2p** |  |
| Species spawning as late as 4^th^ February moon | | | | | | | | | | | | | | |
| *A. divaricata* | 2a,  19w | | **7a,**  **5w,**  **3p** | | **1a,**  **7w,**  **28p** |  | | 1a | | **4a,**  **13w,**  **16p** |  |  |  |  |
| *A. loripes* |  | |  | | **3w,**  **12p** | 7a,  8w | | **20a,**  **8p** | | **3a,**  **19w,**  **29p** |  |  |  |  |
| *A. humilis* |  | |  | | **1w,**  **34p** |  | |  | |  |  |  |  |  |
| *A. secale* |  | |  | | **1a,**  **10p** |  | |  | |  |  |  | 5a |  |
| *A. valencienessi* |  | | 5w | | **4a,**  **6w,**  **20p** |  | |  | |  |  |  |  |  |

In a second set of species, the presence of pigmented eggs in late January implied spawning following the full moon in early February, in at least the Mackay Whitsunday and Wet Tropics regions. For two species, *A. divaricata* (Wet Tropics) and *A. loripes* (Mackay Whitsunday), the presence of pigmented eggs prior to both December and February moons suggest either a protracted spawning period or possible bimodal spawning for some species. As such, most, but not all, *Acropora* spawning was available to sampling over the period of tile deployments.

**Determining the deployment duration for sampling Acroporidae settlement.**

Deployment dates for tiles for 2006-2012 spawning seasons are given in detail in supplementary information Table in S1 Table. For the years 2006-2008, the proportion of settlement recorded on the first deployment compared to second deployment of tiles (Table B) serves to identify the period of peak settlement for Acroporidae, and the variability in timing of spawning between years and reefs within years. In each case, the first deployment of tiles was timed to capture settlement resulting from spawning following full moons in October and November. In 2006, the full moon was earlier in these two months than in 2007 and 2008, and the proportion of settlement occurring over the first deployment was low (mean of 53.5%) compared to 2007 and 2008 (means of 72.5% and 79.4% respectively). These results are consistent with the observation of pigmented eggs remaining in corals after the first deployment in 2006 (Table A). Two exceptions were for Pelican Island and Keppels South in the Fitzroy region (Table B) where in 2006 99.9% and 89.5% (respectively) of settlement occurred on the first tile deployment. In contrast, at Barren Island, which is 12km further offshore than Keppels South, only 4% of the settlement occurred on the first deployment of tiles.

**Table B. Proportion (%) of Acroporidae spat captured by first deployment of tiles**. Date of tile exchange indicated in brackets. Dates of full moons covered by tile deployments provided for each year.

|  |  | Proportion of settlement occurring on 1^st^ deployment tiles (%)  and date of tile exchange | | |
| --- | --- | --- | --- | --- |
| Region | Reef | Full moons 2006  7^th^ Oct, 5^th^ Nov | Full moons 2007  26^th^ Oct, 25^th^ Nov | Full moons 2008  15^th^ Oct, 13^th^ Nov |
| Wet Tropics | Fitzroy Island | 58 %  (28^th^ Nov) | 84 %  (16^th^ Dec) | 81 %  (2^nd^ Dec) |
|  | Frankland Group | 22 %  (29^th^ Nov) | 74 %  (17^th^ Dec) | 63 %  (1^st^ Dec) |
|  | High Island | 8 %  (27^th^ Nov) | 99 %  (16^th^ Dec) | 91 %  (1^st^ Dec) |
| Burdekin | Palms West |  | 55 %  (15^th^ Dec) | 40 %  (3^rd^ Dec) |
|  | Pandora Reef |  | 39 %  (15^th^ Dec) | 72 %  (3^rd^ Dec) |
|  | Geoffrey Bay |  | 98 %  (14^th^ Dec) | 100 %  (4^th^ Dec) |
| Mackay Whitsunday | Double Cone Island | 53 %  (1^st^ Dec) | 79 %  (12^th^ Dec) | 68 %  (6^th^ Dec) |
|  | Daydream Island | 75 %  (1^st^ Dec) | 97 %  (12^th^ Dec) | 93 %  (6^th^ Dec) |
|  | Pine Island | 71 %  (30^th^ Dec) | 95 %  (13^th^ Dec) | 89 %  (7^th^ Dec) |
| Fitzroy | Barren Island | 4 %  (3^rd^ Dec) | 60 %  (11^th^ Dec) | 64 %  (8^th^ Dec) |
|  | Keppels South | 90 %  (3^rd^ Dec) | 59 %  (11^th^ Dec) | 93 %  (8^th^ Dec) |
|  | Pelican Island | 100 %  (4^th^ Dec) | 32 %  (11^th^ Dec) | 100 %  (8^th^ Dec) |

In combination, the generally high proportion of settlement occurring prior to December moons, and the likelihood that corals not spawning in November will spawn in December, suggests that the single deployments from 2009-2011 will have sampled across a similar spawning effort as captured by the earlier dual deployments. We note that neither sampling strategy sampled the late spawning indicated for a subset of species (Table A).

Split spawning was also indicated by settlement results in both deployments for the first three years of the program. For 2006-2008, the proportion of settlement recorded on the first deployment compared to second deployment of tiles (Table B) serves to identify the period of peak settlement for Acroporidae, and the variability in timing of spawning between years and reefs within years. A greater proportion of settlement occurred in the first deployment of the spawning season at most reefs, confirming spawning prior to December moons. The generally high proportion of settlement prior to December moons, and the likelihood that corals not spawning in November would spawn in December, prompted the change in sampling design to single deployments that targeted spawning occurring in late October/ early November and late November/ early December from 2009 onwards (Table B).
