# Supplementary figures and images for: High spatio-temporal variability in Acroporidae settlement to inshore reefs of the Great Barrier Reef
Source: PLoS One. 2019 Jan 30;14(1):e0209771. doi: 10.1371/journal.pone.0209771 (PMC6353100; doi:10.1371/journal.pone.0209771)

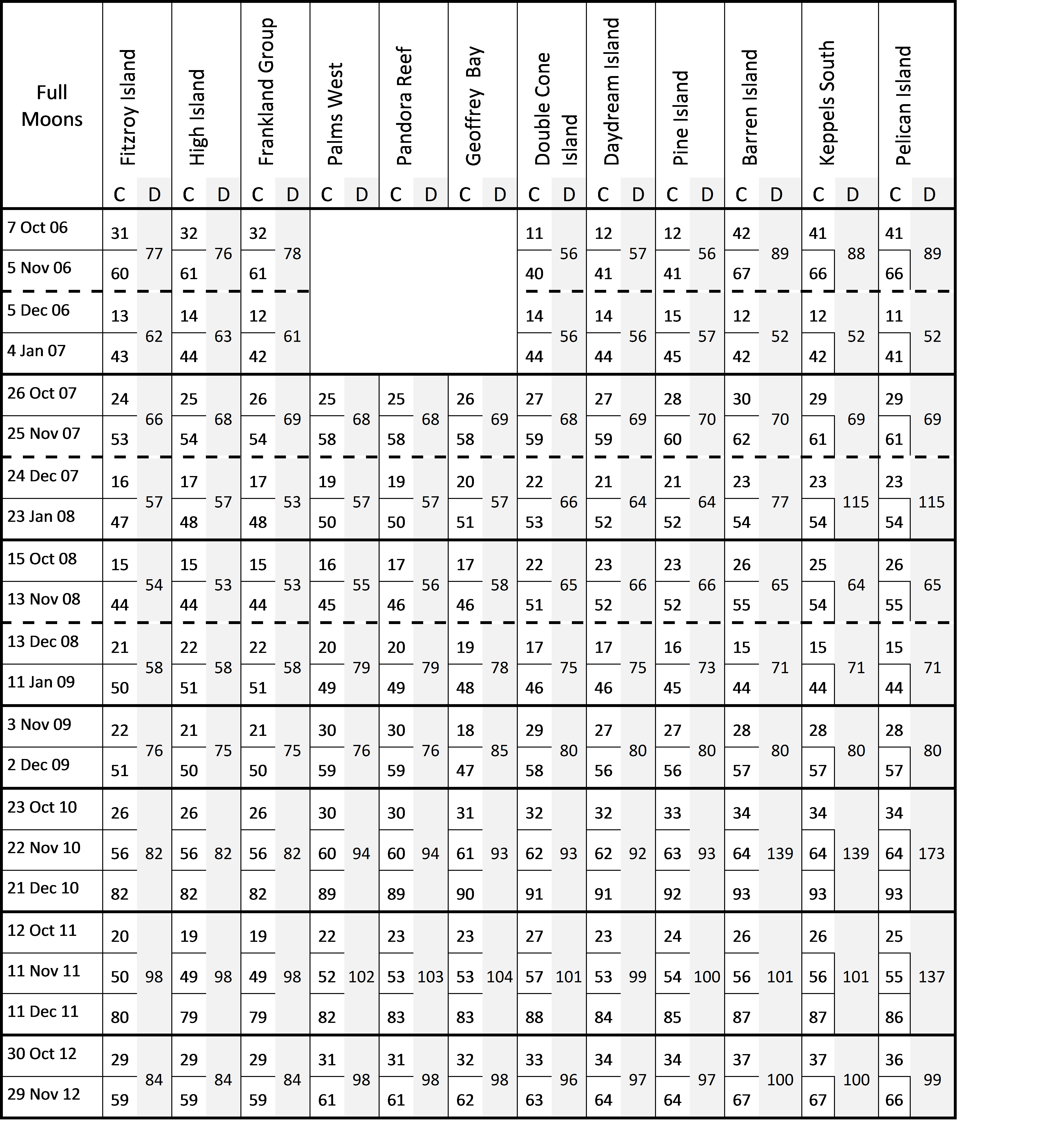

Supplement: S1 Table — The date of the Full Moons during the main spawning season was used to estimate the number of tile conditioning days (C) before competency of settlement of Acroporidae larvae (assumed to be 10 days post-full moon [42]), with the total number of tile deployment days (D) before collection. For years with two tile deployments (2006–2008), the dotted line indicates the limits for conditioning and deployment, when the first set of tiles were retrieved and replaced with a second set of clean tiles. (TIF) [file pone.0209771.s002.tif]
