# Supplementary material for: High spatio-temporal variability in Acroporidae settlement to inshore reefs of the Great Barrier Reef
Source: PLoS One. 2019 Jan 30;14(1):e0209771. doi: 10.1371/journal.pone.0209771 (PMC6353100; doi:10.1371/journal.pone.0209771)
Supplement: S4 Table — Where C = the maximum conditioning period from deployment to +10 days post 2nd (or 3rd if applicable) moon during spawning season, and cover of CCA (%) is an indicator of the level of exposure of coral larvae to CCA among reefs and years. (DOCX) [file pone.0209771.s005.docx]

|  |  | Year | 2006 | | 2007 | | 2008 | | 2009 | 2010 | 2011 | 2012 |
| --- | --- | --- | --- | --- | --- | --- | --- | --- | --- | --- | --- | --- |
| Full Moons | | | **05-Nov 2006** | **04-Jan 2007** | **25-Nov 2007** | **23-Jan 2008** | **13-Nov 2008** | **11-Jan 2009** | **02-Dec 2009** | **21-Dec 2010** | **11-Dec 2011** | **29-Nov 2012** |
| Deployment | | | 1 | 2 | 1 | 2 | 1 | 2 | 1 | 1 | 1 | 1 |
| Fitzroy Island | | C | 60 | 43 | 53 | 47 | 44 | 50 | 51 | 82 | 80 | 59 |
|  |  | CCA | 10.72 | 16.2 | 13.47 | 17.6 | 13.9 | 15.57 | 32.12 | 23.75 | 39.58 | 20.72 |
| High Island | | C | 61 | 44 | 54 | 48 | 44 | 51 | 50 | 82 | 79 | 59 |
|  |  | CCA | 10.83 | 5.15 | 7.07 | 3.56 | 5.42 | 7.96 | 11.52 | 10 | 16.54 | 5.22 |
| Frankland Group | | C | 61 | 42 | 54 | 48 | 44 | 51 | 50 | 82 | 79 | 59 |
|  |  | CCA | 7.86 | 18.87 | 18.31 | 41.3 | 25.31 | 32.53 | 42.86 | 26.6 | 47.01 | 20.82 |
| Palms West | | C |  | | 58 | 50 | 45 | 49 | 59 | 89 | 82 | 61 |
|  |  | CCA |  | | 1.82 | 1.26 | 7.09 | 11.74 | 10.42 | 23.07 | 15.12 | 11.78 |
| Pandora Reef | | C |  | | 58 | 50 | 46 | 49 | 59 | 89 | 83 | 61 |
|  |  | CCA |  | | 24.38 | 35.05 | 26.93 | 30.23 | 39.3 | 39.86 | 32.56 | 27.62 |
| Geoffrey Bay | | C |  | | 58 | 51 | 46 | 48 | 47 | 90 | 83 | 62 |
|  |  | CCA |  | | 17.1 | 25.4 | 14.11 | 9.22 | 23.49 | 29.86 | 13.09 | 21.61 |
| Double Cone Island | | C | 36 | 40 | 59 | 53 | 51 | 46 | 58 | 91 | 88 | 63 |
|  |  | CCA | 6.37 | 6.59 | 3.78 | 4.5 | 9.15 | 9.26 | 10.39 | 17.93 | 7.76 | 8.75 |
| Daydream Island | | C | 37 | 40 | 59 | 52 | 52 | 46 | 56 | 91 | 84 | 64 |
|  |  | CCA | 2.64 | 4.61 | 3.58 | 6.12 | 10.78 | 11.56 | 6.48 | 17.11 | 4.45 | 7.69 |
| Pine Island | | C | 37 | 41 | 60 | 52 | 52 | 45 | 56 | 92 | 85 | 64 |
|  |  | CCA | 4.24 | 5.88 | 6.89 | 9.53 | 7.06 | 5.79 | 10.22 | 16.35 | 5.50 | 4.80 |
| Barren Island | | C | 67 | 38 | 62 | 54 | 55 | 44 | 57 | 93 | 87 | 67 |
|  |  | CCA | 12.22 | 8.24 | 4.68 | 27.93 | 17.19 | 15.74 | 26.63 | 49.38 | 19.81 | 14.37 |
| Keppels South | | C | 66 | 38 | 61 | 54 | 54 | 44 | 57 | 93 | 87 | 67 |
|  |  | CCA | 29.16 | 11.53 | 7.56 | 43.58 | 17.46 | 23.44 | 42.63 | 44.44 | 24.44 | 13.94 |
| Pelican Is | | C | 66 | 65 | 61 | 54 | 55 | 44 | 57 | 93 | 86 | 66 |
|  |  | CCA | 14.82 | 2.21 | 5.62 | 8.53 | 7.11 | 18.88 | 27.48 | 3.4 | 11.01 | 8.81 |
